# The Regulatory Mechanism of 2-Acetyl-1-Pyrroline Biosynthesis in Fragrant Rice (*Oryza sativa* L.) Under Different Soil Moisture Contents

**DOI:** 10.3389/fpls.2021.772728

**Published:** 2021-11-26

**Authors:** Haowen Luo, Meiyang Duan, Leilei Kong, Longxin He, Yulin Chen, Zhimin Wang, Xiangru Tang

**Affiliations:** ^1^State Key Laboratory for Conservation and Utilization of Subtropical Agricultural Bioresources, South China Agricultural University, Guangzhou, China; ^2^Scientific Observing and Experimental Station of Crop Cultivation in South China, Ministry of Agriculture, Guangzhou, China; ^3^Guangzhou Key Laboratory for Science and Technology of Aromatic Rice, Guangzhou, China; ^4^Rice Research Institute, Guangdong Academy of Agricultural Sciences/Guangdong Key Laboratory of New Technology in Rice Breeding/Guangdong Rice Engineering Laboratory, Guangzhou, China; ^5^College of Natural Resources and Environment, College of Agriculture, South China Agricultural University, Guangzhou, China; ^6^College of Engineering, South China Agricultural University, Guangzhou, China

**Keywords:** fragrant rice, 2-acetyl-1-pyrroline, gene expression, enzymes, proline, soil moisture

## Abstract

2-acetyl-1-pyrroline (2-AP) is the key compound of rice aroma. However, the responses of 2-AP biosynthesis in fragrant rice under different soil moisture and the corresponding mechanism are little known. The present study evaluated the effects of different soil moisture on 2-AP biosynthesis through a pot experiment. Four soil moisture contents, that is, 50% (SM50), 40% (SM40), 30% (SM30), and 20% (SM20), were adopted, and SM50 treatment was taken as control. The pots were weighed and watered to maintain the corresponding soil moisture content. The results showed no significant difference in growth parameters (plant height, stem diameter, and plant dry weight) among all treatments. Compared with SM50, SM40, SM30, and SM20 treatments significantly (*p*<0.05) increased 2-AP content by 32.81, 23.18, and 53.12%, respectively. Between 20 to 90% higher proline content was observed in SM40, SM30, and SM20 treatments than in SM50. Enzymes including proline dehydrogenase, ornithine transaminase, and 1-pyrroline-5-carboxylate synthetase exhibited lower activities with soil moisture declined. Higher diamine oxidase activity was observed in SM40, SM30, and SM20 treatments compared with SM50, and real-time PCR analyses showed that transcript level of *DAO1* was greatly increased under low soil moisture treatments, especially in SM20 treatment. Transcript levels of *PRODH*, *DAO2*, *DAO4*, *DAO5*, *OAT*, *P5CS1*, and *P5CS2* decreased or maintained in SM40, SM30, and SM20 treatments compared with SM50. We deduced that low soil moisture content enhanced 2-AP biosynthesis mainly by upregulating the expression of *DAO1* to promote the conversion from putrescine to 2-AP.

## Introduction

**Figure 1:**
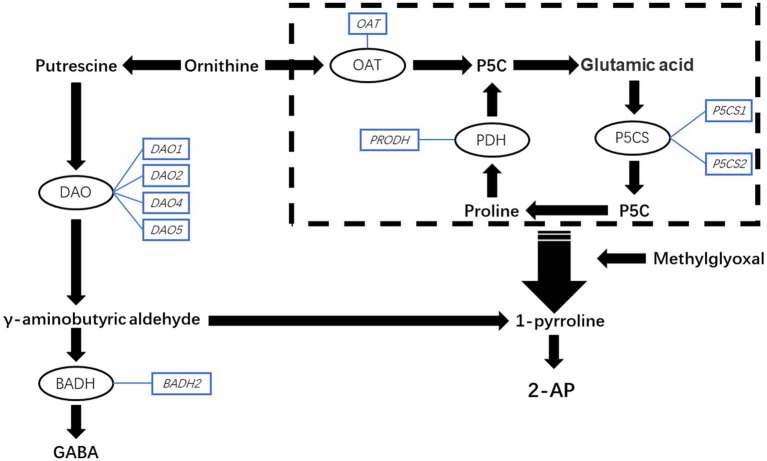
The schematic representation of the potential pathway of 2-AP biosynthesis in fragrant rice. GABA, γ-aminobutyric acid; P5CS, 1-pyrroline-5-carboxylate synthetase; PDH, proline dehydrogenase; OAT, ornithine transaminase; BADH, betaine aldehyde dehydrogenase; DAO, diamine oxidase; P5C, pyrroline-5-carboxylic acid.

Fragrant rice is famous for its special aroma in the world (Dumitrascu et al., 2021; Moogi et al., 2021), and 2-acetyl-1-pyrroline (2-AP) has been identified as the key and main component of that aroma in recent years (Jezussek et al., 2002; Mahattanatawee and Rouseff, 2014; Poonlaphdecha et al., 2016). Over the past two decades, scientists have made attempts to understand the biosynthetic mechanism of 2-AP. The studies by Yoshihashi et al. (2002) and Poonlaphdecha et al. (2016) revealed that proline, glutamic acid, ornithine, and 1-pyrroline are the important precursors of 2-AP. Kaikavoosi et al. (2015) found that pyrroline-5-carboxylate synthetase (P5CS) and the genes encoding it are involved in 2-AP biosynthesis. The study by Chen et al. (2008) found that the expression of gene *BADH2* is important for regulating 2-AP content in fragrant rice because it inhibits 2-AP production by encoding betaine aldehyde dehydrogenase (BADH), which prevents the conversion from γ-aminobutyric aldehyde to 1-pyrroline by turning γ-aminobutyric aldehyde into γ-aminobutyric acid (GABA). The potential 2-AP biosynthesis in fragrant rice is presented in Figure 1.

The irrigation regime could substantially influence the 2-AP content. Mo et al. (2019a) indicated that moderate drought management (soil water potential was between −15 and −25kPa) at the booting stage could remarkably increase grain 2-AP content. Deng et al. (2018) demonstrated that mild drought treatment (the soil water potential was −25kPa) during the grain-filling stage also increased grain 2-AP. An earlier study also showed that different water regimes at tillering stage induced regulations in levels of 2-AP and its precursors (Li et al., 2021). Those studies found that drought treatment could cause 2-AP increment, accompanied by upregulation of proline content, and thus, they deduced that drought increased 2-AP by increasing proline content and thus promoting the conversion from proline to 2-AP. However, this theory lacks data support at the molecular level, and more studies should be conducted at the physiological level. In 2018, the study by Bao et al. (2018) showed that alternate wetting and drying irrigation during the grain-filling stage greatly increased grain 2-AP content by upregulating the proline content, and the activity of proline dehydrogenase (PDH) and expression of *PRODH* were also upregulated. Therefore, they believed that increased 2-AP was converted from accumulated proline under the irrigation strategy. However, alternate wetting and drying irrigation includes dehydration and recovery parts, while recovery would induce proline degradation as reported by a previous study (Foster et al., 2015). Those studies cannot elaborate on the mechanism of 2-AP biosynthesis under different soil water conditions.

Water management would change and control the soil moisture, while many physiological activities of crops are sensitive to the change of soil moisture. The research of Thai et al. (2019) revealed that as soil moisture declined, photosynthetic parameters of sugarcane decreased to some degree, and a soil moisture level of 10% might be the critical point beyond which irrigation results in photosynthetic disorders. Zhang et al. (2021) demonstrated that the degree of soil drying substantially influenced the yield formation of rice. Farrell et al. (2017) indicated that plant species vary relative water content (RWC) and water potential at turgor loss point and concomitant drought sensitivity. The studies on soil moisture-induced physiological and biochemical responses are important to understand the regulatory mechanism of water management on 2-AP biosynthesis and propose practical irrigation strategies to achieve the goal of high yield and high aroma in fragrant rice production. Little is known; however, the response of 2-AP level to different soil moisture and the related mechanism at the physiological and molecular levels has rarely been reported.

The objectives of this study were to evaluate the effects of different soil moisture content on 2-AP biosynthesis and the mechanism behind it. Besides 2-AP, eight compounds, nine genes, and five enzyme activities involved in 2-AP biosynthesis were determined under different soil moisture treatments. Such a study would provide new and further information about the physiological mechanism of 2-AP biosynthesis in fragrant rice.

## Materials and Methods

### Plant Materials, Growth Condition, and Experimental Design

The pot experiment was conducted in an artificial climate chamber in the College of Agriculture, South China Agricultural University, Guangzhou, China. The parameters of the chambers were set as 28/25°C day/night temperature and 70% air relative humidity. The seeds of a fragrant rice cultivar, *Xiangyaxiangzhan*, which was widely planted in South China, were used as plant materials in the experiment. After germination, rice seeds were sowed in hydroponic boxes, and then, the rice seedlings were allowed to grow in a commercial hydroponic solution (Hyponex, Osaka, Japan) under controlled conditions (temperature: 26±2°C) according to the methods of Mostofa et al. (2020). Then, 16-day-old healthy seedlings were transplanted into pretreated and soil-contained pots with nine seedlings per pot. Each pot was filled with 500g dry soil before, and 10 pots were used for each treatment. Before transplanting, each pot was watered to reach the corresponding soil moisture, and four soil moisture contents were set as 20% (SM20), 30% (SM30), 40% (SM40), and 50% (SM), respectively. The soil moisture percentage means the proportion of water weight to dry soil weight. The pots were weighed and watered every 8h to maintain the corresponding soil moisture. The experimental soil was sandy loam containing 11.85gkg^−1^ organic matter, 0.46gkg^−1^ total nitrogen, 0.31gkg^−1^ total phosphorus, 17.59gkg^−1^ total potassium, 10.05mgkg^−1^ available phosphate, 27.83mgkg^−1^ available nitrogen, 52.43mgkg^−1^ available potassium, and 6.08 pH. The saturated soil moisture was 53.68% (proportion of water weight to dry soil weight).

### Measurements of Plant Growth Parameters and Sampling

Ten days after receiving soil moisture treatments, nine seedlings were randomly selected and taken from each treatment to immediately measure the fresh weight, plant height, and stem width. Then, the seedlings were subjected to oven-drying at 80°C for 48h to determine the dry weight, and RWC was calculated using the methods of Mostofa et al. (2020). To determine metabolite contents and enzyme activities, three pots were selected as three biological replicates in each treatment. The fresh leaves were sampled and stored at −80°C, and the determination was repeated thrice for the individual parameters. For gene expression analysis, another three pots were selected as three biological replicates in each treatment, and about 1.00g of fresh leaves of each pot was separately sampled and stored at −80°C for quantitative real-time polymerase chain reaction (qRT-PCR).

Determination of 2-AP, 1-pyrroline, proline, methylglyoxal, glutamic acid, soluble protein, soluble sugar, GABA, and P5C contents.

The 2-AP content was determined with synchronization distillation and extraction method (SDE) combined with GCMS-QP 2010 Plus (Shimadzu Corporation, Japan) according to the methods of Bao et al. (2018). The final 2-AP content was expressed as μgkg^−1^ fresh weight (FW).

The determination of 1-pyrroline content was carried out with the methods of Hill (1967). The reaction system contained 1ml of 0.01M 2-amino benzaldehyde (in 0.02M phosphate buffer, pH 7.0), 1ml of 0.2M phosphate buffer, and 1ml of distilled water. The 1-pyrroline concentration was calculated with the molar extinction coefficient (ε=1860cm^−l^) after the absorbance was read at 430nm. The final 1-pyrroline content was expressed as μmolg^−1^ FW.

The determination of glutamic acid content was carried out according to Wang et al. (2005). After reacting with 0.5% 2,2-dihydroxyindane-1,3-dione at boiling water bath for 20min, the absorbance was read at 569nm, and the concentration was calculated from a standard curve (the glutamic acid standard curve was made using L-glutamic acid (CAS: 56-86-0) as standard compound). The final glutamic acid content was expressed as mgg^−1^ FW.

The determination of proline content was carried out with the method of Bates et al. (1973). The sample (about 0.1g) was homogenized in 2ml of 3% sulfosalicylic acid and boiled for 10min in water bath. The 0.5ml of the filtrate was mixed with ninhydrin reagent (0.5ml) and glacial acetic acid (0.5ml). The reaction mixture was again placed in boiling water bath for 30min and then extracted with 1.5ml of toluene. The absorbance was measured at 520nm, and the concentration was calculated from a standard curve. The final proline content was expressed as μgg^−1^ FW.

The determination of methylglyoxal content was carried out with the methods of Banu et al. (2010). In a total volume of 1ml, 250μl of 1,2-diaminobenzene, 100μl of perchloric acid, and 650μl of the neutralized supernatant were added in that order. Then, the absorbance of the derivative was read at 336nm, and the concentration was calculated from a standard curve. The final methylglyoxal content was expressed as mgg^−1^ FW.

The determination of soluble protein content was carried out according to Mostofa et al. (2020). 0.2ml of extracting solution reacted with 1.0ml of Coomassie Brilliant Blue G250 Reagent at room temperature for 20min. The absorbance of the derivative was read at 595nm, and the concentration was calculated from a standard curve [the protein a standard curve was made using bovine serum albumin (CAS: 9048-46-8) as standard compound]. The final soluble protein content was expressed as mgg^−1^ FW.

The determination of soluble sugar content was carried out according to Mostofa et al. (2020). About 0.1g sample was extracted using 10ml of distilled water and boiled for 20min. The 0.2ml of the filtrate was mixed with 1.0ml anthrone. The reaction mixture was again placed in a boiling water bath for 20min. The absorbance was measured at 620nm. The concentration was calculated from a standard curve (the sugar standard curves were made using D-glucose anhydrous (CAS: 50-99-7) as standard compound). The final soluble sugar content was expressed as μgg^−1^ FW.

The determination of GABA content was carried out with the methods of Bao et al. (2018). The extraction solution contained 60% ethanol, 60mM lanthanum chloride, and 1M KOH. The supernatant was added to 0.2 M borate buffer (pH 10.0) and 6% phenol solution, and sodium hypochlorite (available chlorine, 10%) was added while shaking. The absorbance was measured at 645nm, and the concentration was calculated from a standard curve. The final GABA content was expressed as μgg^−1^ FW.

The determination of P5C content was carried out with the methods of Bao et al. (2018). The extraction solution contained 50mM Tris–HCl (pH 8.0), 10% glycerol, 1% triton-100, and 1% β-mercaptoethanol. The supernatant was added to a mixture containing 10% trichloroacetic acid (TCA) and 40mM γ-aminobenzaldehyde. The P5C concentration was calculated with the molar extinction coefficient (ε=2.58mMcm^-l^) after the absorbance was read at 430nm. The final P5C content was expressed as μmolg^−1^ FW.

### Assay of Enzymes (P5CS, PDH, Ornithine Transaminase, Betaine Aldehyde Dehydrogenase, and Diamine Oxidase) Activities

P5CS activity was determined according to the methods of Sánchez et al. (2002). The 0.5ml of reaction mixture volume contained 50mmol/L Tri-HCl pH 7.0, 50mmol/L L-glutamate, 20mmol/L MgCl_2_, 10mmol/L ATP, and 100mmol/L hydroxamate-HCl. The absorbance was read at 535nm after the reaction. The determination of PDH activity was carried out according to Ncube et al. (2013) methods, and the absorbance was read at 440nm after the reaction. The determination of OAT activity was carried out according to the methods of Deng et al. (2018). The reaction mixture contained 100mM potassium phosphate buffer (pH 8.0), 1mM pyridoxal-5-phosphate, 50mM ornithine, 20mM α-ketoglutarate, and the enzyme extract (0.1ml). After incubation of the assay mixture for 30min, the absorbance was read at 440nm. The determination of BADH activity was carried out according to Yan et al. (2012), and the activity was both expressed as Ug^−1^ h^−1^. DAO activity was determined according to the methods of Yang et al. (2011). Reaction solutions (2.9ml) contained 2.0ml of 70mmol/l sodium phosphate buffer, 0.5ml of crude enzyme extracts, 0.1ml of horseradish peroxidase, and 0.2ml of 4-aminoantipyrine/N,N-dimethylaniline. The reaction was initiated by adding 0.1ml of putrescine, and the absorbance was read at 555nm.

### Real-Time Quantitative RT-PCR

Total RNA was extracted using the HiPure Plant RNA Mini Kit (Magen, Guangzhou, China). The quality and quantity of RNA were assessed by NanoDrop 2000. The Hiscript II QRT SuperMix for qPCR (+gDNAwiper; Vazyme, Nanjing, China) synthesized cDNA from total RNA. Real-time quantitative RT-PCR (qRT-PCR) was conducted in the CFX96 real-time PCR System (Bio-Rad, Hercules, CA, United States). Actin was used as an internal reference gene. Primers used for qRT-PCR are listed in Table 1. All primers were designed using the software tool Primer 5.

**Table 1:** Primer sequences of genes encoding enzymes involved in 2-AP biosynthesis.

Gene name	Accession no.	Primer sequences
*Proline dehydrogenase (PRODH)*	AP014966.1	F 5'-TCATCAGACGAGCAGAGGAGAACAGG-3' R 5'-CCCAGCATTGCAGCCTTGAACC-3'
*Pyrroline-5-carboxylic acid synthetase1 (P5CS1)*	AP014961.1	F 5'-TTTTGAGTCCCGACCTG-3' R 5'-TTCACCAACATTACGAGGA-3'
*Pyrroline-5-carboxylic acid synthetase2 (P5CS2)*	AP014957.1	F 5'-GAGGTTGGCATAAGCACAG-3' R 5'-CTCCCTTGTCGCCGTTC-3'
*Ornithine aminotransferase (OAT)*	AP014959.1	F 5'-GCCCTTGGTGCTGGAGTA-3' R 5'-AGCCCTTTCAACGAGACCTT-3'
*Diamine oxidase1 (DAO1)*	AP014962.1	F 5'-ATGGCACCCGGAACTCTTTC-3' R 5'-GCCTCAGTCTCGGCAACCTC-3'
*Diamine oxidase2 (DAO2)*	AP014960.1	F 5'-TCGTTCGCATCAAGGTTGG-3' R 5'-TCAGACAGAAGGGTGCCGTA-3'
*Diamine oxidase4 (DAO4)*	AP014960.1	F 5'-TGGCAAGATAGAAGCAGAAGT-3' R 5'-GTCCATACGGGCAACAAA-3'
*Diamine oxidase5(DAO5)*	AP014958.1	F 5'-TGGCAAGATAGAAGCAGAA-3' R 5'-TCCATACGGGCAACAAA-3'
*Betaine aldehyde dehydrogenase (BADH2)*	AB09683	F 5'-GGTTGGTCTTCCTTCAGGTGTGC-3' R 5'-CATCAACATCATCAAACACCACTAT-3'

### Data Analysis

All the obtained data were subjected to a one-way analysis of variance (ANOVA) using Statistix 8.1 (Analytical Software, Tallahassee, FL, United States). Statistix 8.1 was also used to perform correlation analysis, and the heatmap for the investigated parameters was established with Microsoft Excel. The differences among means were separated using the least significant (LSD) test at the 5% probability level. Sigma Plot 9.0 (Systat Software Inc., San Jose, CA, United States) was used to make figures.

## Results

### 2-AP Content

**Figure 2:**
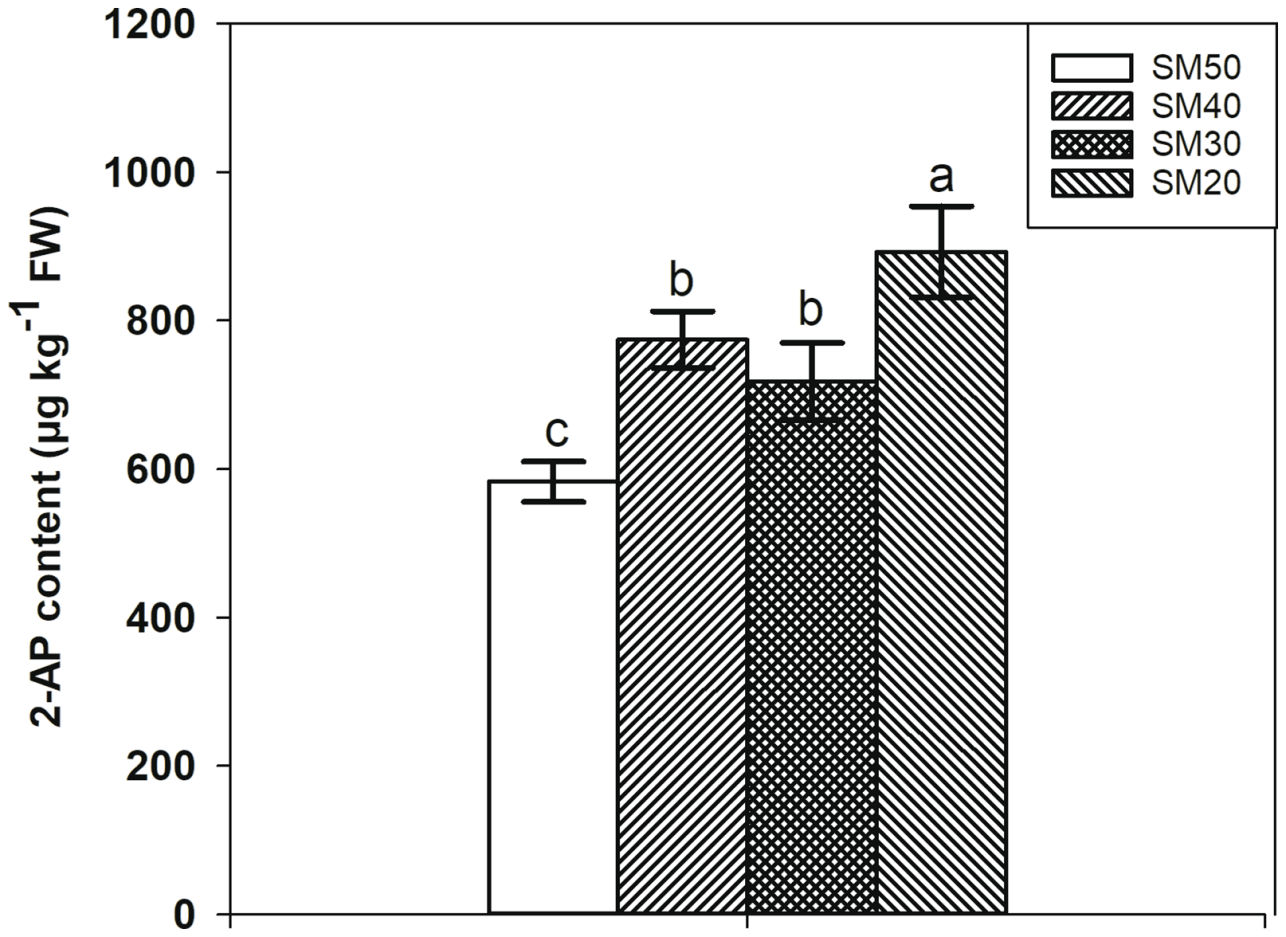
Increase of 2AP content in fragrant rice with reduction of soil moisture. Each column represents the mean of three data±standard error (*n*=3). Bars sharing a common letter do not differ significantly at *p*<0.05.

The 2-AP content under different soil moisture is shown in Figure 2. The lowest 2-AP content was recorded in the control (SM50 treatment), while the highest 2-AP content was recorded in SM20 treatment. Compared with SM50, SM40, SM30, and SM20 treatments significantly (*p*<0.05) increased 2-AP content by 32.81, 23.18, and 53.12%, respectively. There was no significant difference between SM40 and SM30 treatments.

### Growth Parameters

The plant height, stem width, fresh weight, dry weight, and relative water content of fragrant rice under different soil moisture are shown in Table 2. There was no significant (*p*<0.05) difference among the four treatments in plant height, stem width, and dry weight. Compared with SM50, SM40, SM30, and SM20 treatments significantly decreased the fresh weight of fragrant rice by 8.82, 10.29, and 9.17%, respectively. The differences in fresh weight among SM40, SM30, and SM20 treatments were not significant. The relative water content was between 75 and 77%, with a slight reduction with the decline of the soil moisture.

**Table 2:** Effect of different soil moisture contents on growth parameters of fragrant rice plants.

Treatment	Plant height (cm)	Stem width (mm)	Fresh weight (mg plant-1)	Dry weight (mg plant-1)	Relative water content (%)
SM50	20.05 ± 1.03a	2.18 ± 0.09a	188.89 ± 14.35a	42.33 ± 3.21a	77.51 ± 1.99a
SM40	20.89 ± 1.22a	2.19 ± 0.15a	172.22 ± 13.84b	41.00 ± 2.83a	76.06 ± 2.46ab
SM30	20.07 ± 1.11a	2.27 ± 0.11a	169.44 ± 9.98b	40.89 ± 3.48a	75.79 ± 2.62ab
SM20	20.22 ± 1.33a	2.24 ± 0.12a	171.56 ± 13.56b	42.11 ± 2.85a	75.37 ± 1.90b

Data represent the mean of five data±standard error. Different letter above the table indicates difference at *p*<0.05 by LSD tests.

### 1-Pyrroline, Methylglyoxal, Glutamic Acid, Proline, GABA, P5C Contents, Soluble Protein, and Soluble Sugar Contents

**Figure 3:**
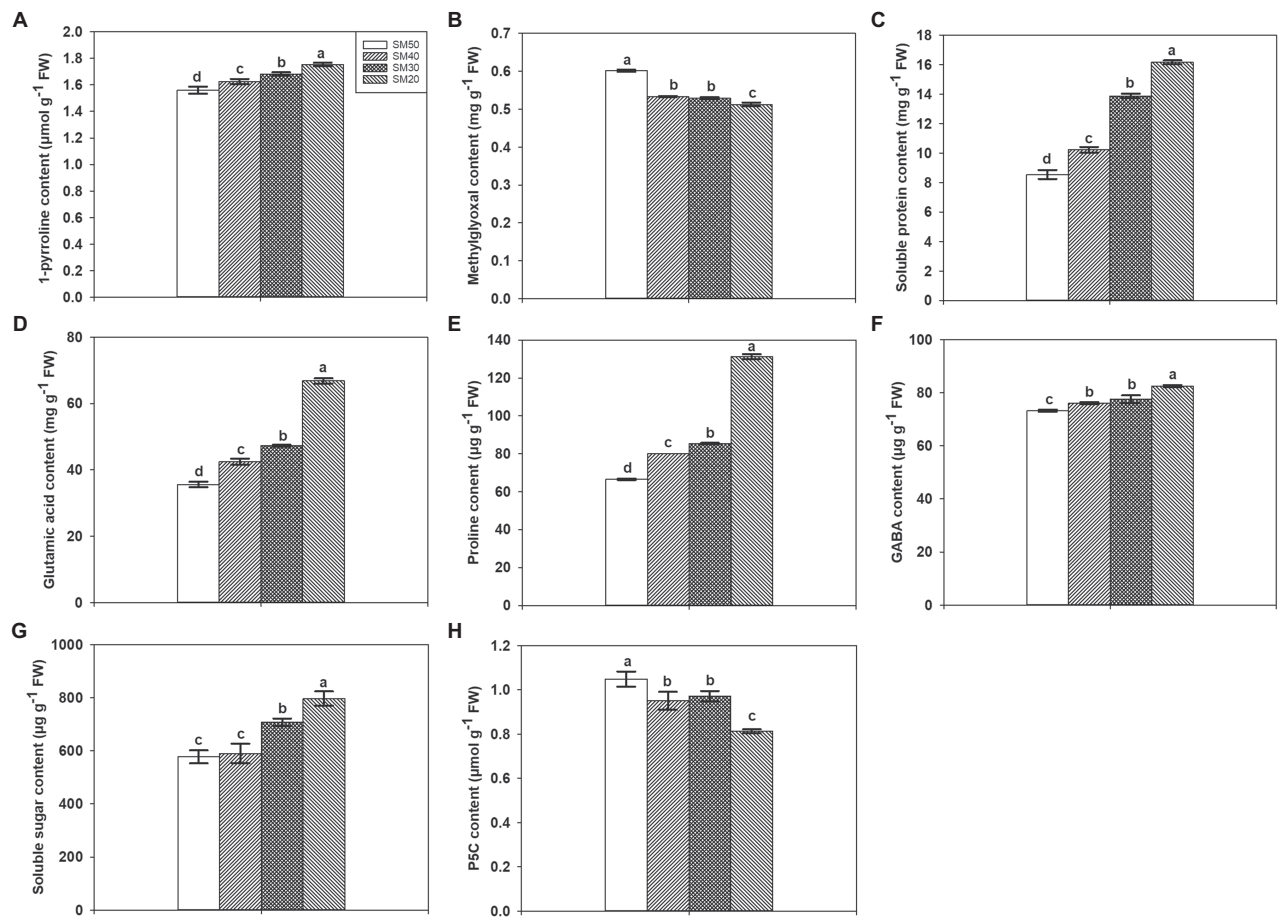
Effects of different soil moisture contents on 1-pyrroline **(A)**, methylglyoxal **(B)**, soluble protein **(C)**, glutamic acid **(D)**, proline **(E)**, GABA **(F)**, soluble sugar **(G)**, and P5C **(H)** contents in fragrant rice. Each column represents the mean of three data±standard error (*n*=3). Bars sharing a common letter do not differ significantly at (*p*<0.05).

Different soil moisture substantially affected the contents of many biochemical substances (Figure 3). Compared with SM50 treatment, SM40, SM30, and SM20 treatments significantly (*p*<0.05) increased 1-pyrroline content by 4.22, 7.83, and 12.46%. Higher methylglyoxal contents were recorded in SM40, SM30, and SM20 treatments compared with SM50. With the decrease of soil moisture from 50 to 20%, soluble protein, glutamic acid, and proline contents significantly increased by 19.58–88.99%, 19.40–87.84%, and 20.46–97.19%, respectively. Compared with SM50, SM30 and SM20 treatments significantly increased GABA and soluble sugar contents. Lower P5C contents were recorded in SM40, SM30, and SM20 treatments than in SM50.

### Expression Levels of Genes Related to 2-AP Biosynthesis

**Figure 4:**
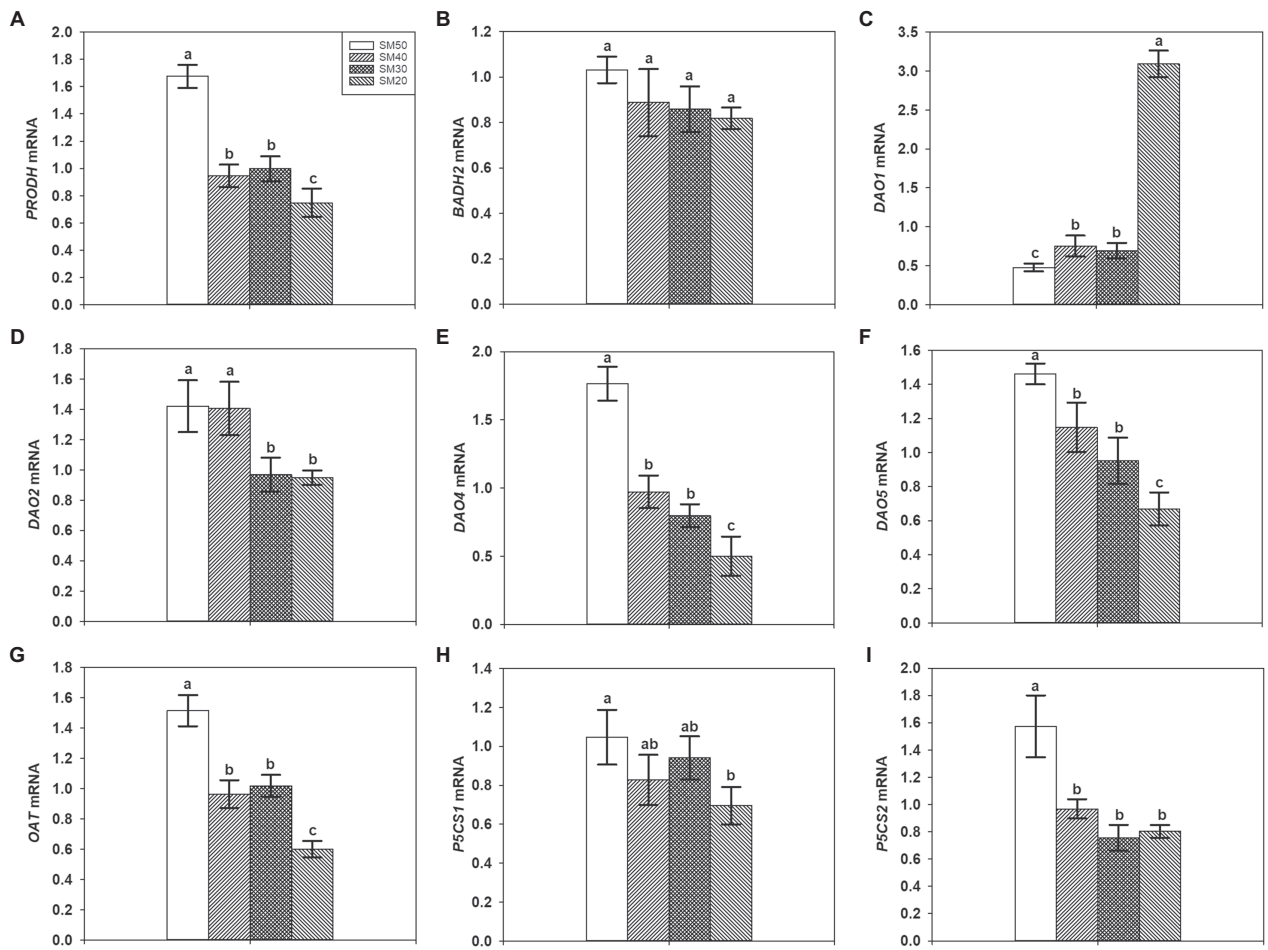
Effects of different soil moisture contents on expression levels of *PRODH* **(A)**, *BADH2* **(B)**, *DAO1* **(C)**, *DAO2* **(D)**, *DAO4* **(E)**, *DAO5* **(F)**, *OAT* **(G)**, *P5CS1* **(H)**, and *P5CS2* **(I)** in fragrant rice. Each column represents the mean of three data±standard error (*n*=3). Bars sharing a common letter do not differ significantly at (*p*<0.05).

Figure 4 shows the transcript levels of *PRODH*, *BADH2*, *DAO1*, *DAO2*, *DAO4*, *DAO5*, *OAT*, *P5CS1*, and *P5CS2* under different soil moisture. Compared with SM50, SM40, SM30, and SM20 treatments significantly (*p*<0.05) decreased transcript levels of *PRODH*, *DAO4*, *DAO5*, and *OAT* by 21.45–44.94%, 32.74–54.85%, and 54.18–71.70%, respectively. The transcript level of *BADH2* decreased with the decline of soil moisture, but the differences among all treatments are not significant. 58.19, 45.05, and 548.05% higher transcript levels of *DAO1* were recorded in SM40, SM30, and SM20 treatments than in SM50, respectively. 31.74 and 33.22% lower transcript levels of *DAO2* were recorded in SM30 and SM20 treatments than in SM50, respectively. Compared with SM50, SM40, SM30, and SM20 treatments significantly decreased transcript levels of *P5CS2*. There was no significant difference among SM50, SM40, and SM30 treatments in transcript level of *P5CS1*, but 33.54% lower transcript level was recorded in SM20 treatment than in SM50 treatment.

### PDH, P5CS, BADH, OAT, and DAO Activities

**Figure 5:**
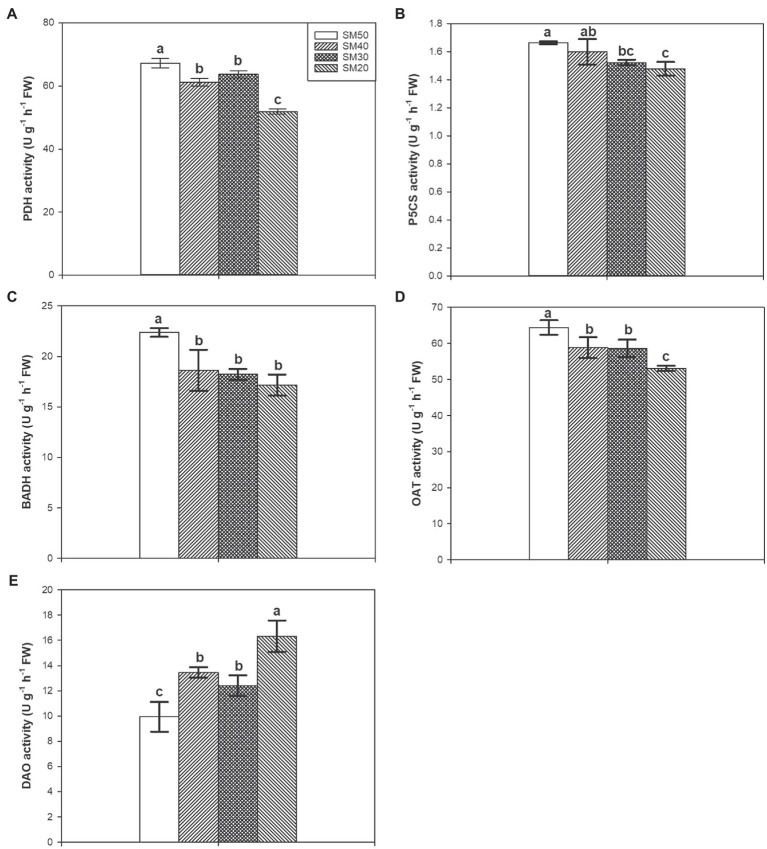
Effects of different soil moisture contents on activities of PDH **(A)**, P5CS **(B)**, BADH **(C)**, OAT **(D)**, and DAO **(E)** in fragrant rice. Each column represents the mean of three data±standard error (*n*=3). Bars sharing a common letter do not differ significantly at (*p*<0.05).

Different soil moisture substantially regulated PDH, P5CS, BADH, OAT, and DAO activities (Figure 5). Compared with SM50, SM40, SM30, and SM20 treatments significantly (*p*<0.05) reduced PDH activity by 8.98, 5.26, and 22.83%, respectively. There was no significant difference between SM50 treatment and SM40 treatment in P5CS activity but SM30 and SM20 treatments significantly reduced P5CS activity compared with SM50. Lower BADH and OAT activities were recorded in SM40, SM30, and SM20 treatments than in SM50. 35.56, 24.66, and 64.24% higher DAO activities were recorded in SM40, SM30, and SM20 treatments than in SM50, respectively.

### Correlation Analysis

**Figure 6:**
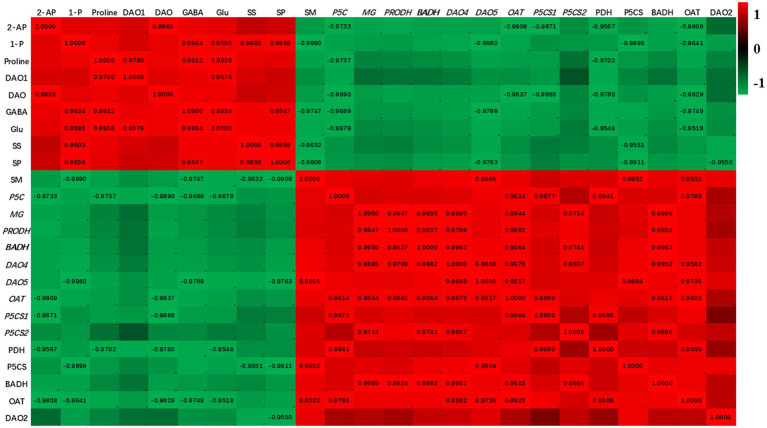
The heatmap for the investigated parameters. Values are fit constants (only show when *p*<0.05). 2-AP, 2-acetyl-1-pyrroline; SM, soil moisture content; 1-P, 1-pyrroline content; MG, methylglyoxal content; SP, soluble sugar content; Glu, glutamic acid content: SS, soluble sugar content; P5C, pyrroline-5-carboxylic acid; PDH, proline dehydrogenase; P5CS, pyrroline-5-carboxylate synthetase; BADH, betaine aldehyde dehydrogenase; OAT, ornithine transaminase; DAO, diamine oxidase.

The heat map of the correlations among the enzymes activities, biochemical substances contents, genes expressions, and 2-AP content is presented in Figure 6. 2-AP content in fragrant rice was negatively correlated with soil moisture content. There were positive correlations among 2-AP content, the transcript level of *DAO1*, and DAO activity. Glutamic acid, proline, and GABA contents negatively correlated with soil moisture. The transcript levels of *PRODH*, *BADH2*, *DAO2*, *DAO4*, *DAO5*, *OAT*, *P5CS1*, and *P5CS2* negatively correlated with 2-AP but positively correlated with soil moisture content, and similar trends were observed in activities of PDH, P5CS, BADH, and OAT.

## Discussion

The rice aroma with 2-AP as the key compound is a highly heritable trait governed by genes, although rice varieties with aromatic properties differ genetically (Okpala et al., 2019). The present study revealed the effects of different soil moisture contents on 2-AP biosynthesis in fragrant rice. Our results showed that the lower the soil moisture, the higher the 2-AP. Similar results were also reported by Deng et al. (2018), who demonstrated that a decline of soil water potential could remarkably increase 2-AP content. Our results also showed that the low soil moisture would not inhibit the growth of fragrant rice when it is not less than 20% for 10days according to the determined growth parameters (plant height, stem width, and dry weight). These results were consistent with previous studies, which showed that intermittent drought would not affect rice growth or reduce grain yield (Wang et al., 2016; Zhang et al., 2021). Our results were consistent with the study by Mo et al. (2019b), who indicated that suitable drought treatment could increase 2-AP content without inhibiting the productivity of fragrant rice.

Our study observed that high 2-AP induced by low soil moisture accompanied by proline accumulation. Our results agreed with the study by Mo et al. (2019a), who showed that decreased water potential increased both proline and 2-AP contents in fragrant rice and demonstrated that the increased 2-AP was attributed to the enhanced conversion from proline to 2-AP. However, this theory contrasts with the results of the present study. Although the proline content was increased in low soil moisture treatments (SM40, SM30, and SM20, compared with SM50), PDH, which plays a crucial part in proline degradation (Huang et al., 2007; Luo et al., 2021), presented an opposite trend in its activity. The results of our study were consistent with the study by Amist and Singh (2017), which showed that PDH activity in wheat plants substantially declined under drought stress. The decline of ProDH activity could be attributed to the transcript reduction. In addition, P5CS and OAT, which are enzymes involved in proline metabolism and responsible for converting glutamic acid and ornithine to proline (Hien et al., 2003; Hinge et al., 2016), displayed lower activities under low soil moisture treatments. Real-time PCR analyses also showed that the expression of *OAT*, *P5CS1*, and *P5CS2* decreased in low soil moisture treatments. Therefore, we deduced that the increase of proline is mainly due to ProDH inhibition. There is possible that P5CS and OAT were induced in earlier times of the soil moisture conditions, and part of the increased proline came from the protein degradation (Trovato et al., 2019). Either way, the decline of PDH activity induced by reducing *PRODH* expression indicated that the proline pathway is not the major way to biosynthesize 2-AP in fragrant rice under low soil moisture.

Moreover, glutamic acid is the precursor of both proline and 2-AP (Yoshihashi et al., 2002; Szabados and Savouré, 2010). In our study, the contents of glutamic acid significantly increased with the decline of soil moisture. This result was inconsistent with the study by Woodrow et al. (2017), who indicated that glutamic acid decreased when proline increased under stress conditions. The present study showed that low soil moisture significantly increased glutamic acid by inhibiting its degradation. The P5CS and its encoded gene (*P5CS1*, *P5CS2*), which correspond to the degradation of glutamic acid, exhibited lower activity and transcript levels under low soil moisture. The differences between our results and previous reports could be explained by different plant species and the strength and duration of drought. The drought condition in the present study did not cause real stress because there was no substant inhibition in rice growth or reduction of RWC. We deduced that the proline accumulation induced the decline of P5CS activity and expression of genes, thus causing the accumulation of glutamic acid. More studies need to be conducted to investigate the effects of soil moisture on glutamate biosynthesis in rice.

The results of our study contrasted with the study by Bao et al. (2018), which showed that alternate wetting and drying treatment increased 2-AP content in fragrant rice by enhancing PDH activity and improving the expression of *PRODH*. Although alternate wetting and drying irrigation would periodically decrease soil moisture, it has the part of recovery, and the compensation effect often occurs after the recovery. The previous study has found that recovery after the drought treatment would induce proline degradation, and it was attributed to the enhancement of PDH activity (Kiyosue et al., 1996; Foster et al., 2015). Thus, we deduced that the 2-AP biosynthesis in fragrant rice mainly depended on the proline pathway only during the recovery phase (wet period) in alternate wetting and drying irrigation. However, the 2-AP biosynthesis relies more on other pathways under drought conditions.

Besides the proline pathway, ornithine would convert to putrescine, then to γ-aminobutyric aldehyde, and then to 1-pyrroline synthesizes 2-AP in fragrant rice (Wakte et al., 2017; Bao et al., 2018). DAO transformed putrescine into γ-aminobutyric aldehyde, which further cyclizes spontaneously to 1-pyrroline, the limiting precursor of 2-AP (Struve and Christophersen, 2003; Ghosh and Roychoudhury, 2017). Our study noted the enhanced DAO activity and increased 1-pyrroline content under low soil moisture treatments. The transcript level of *DAO1* was greatly improved, especially in SM20 treatment, although the transcript levels of *DAO2*, *DAO4*, and *DAO5* presented decreased trends with the decline of soil moisture. The correlation analysis showed that increased 2-AP positively correlated with both *DAO1* expression and DAO activity. Hence, we deduced that increased 2-AP content under low soil moisture could be attributed to the DAO pathway and mainly regulated by the expression of *DAO1*.

In addition, rice growth could be divided into several stages, including seedling, tillering, booting, heading, grain-filling, and maturity. There is possible that the 2-AP synthesis pathway is changing with development and organ. Furthermore, during the heading to maturity stage, the dry weight and some biochemical substances would be transported from vegetative organs to reproductive organs, that is, grains (Liu et al., 2019). It is unknown that whether the 2-AP and its precursors would be transported inside fragrant rice. Thus, more studies need to be conducted to investigate the 2-AP biosynthesis and its response to soil moisture under different growing phases.

**Figure 7:**
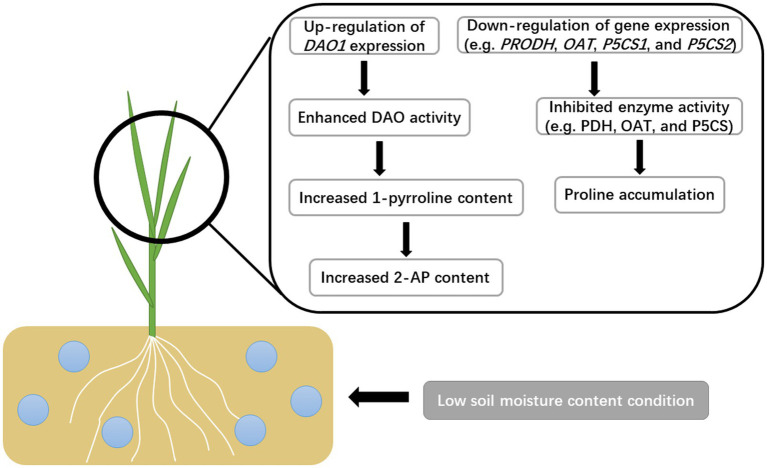
The potential mechanism of soil moisture in regulating the 2-AP biosynthesis in fragrant rice.

In conclusion, the present study indicated that enhanced 2-AP production in fragrant rice plants under low soil moisture was mainly through the upregulation of *DAO1* expression to promote the conversion from putrescine to 2-AP (Figure 7), while the proline pathway was inhibited. In fragrant rice production, applying a moderate water deficit that does not significantly reduce the RWC, growth, and yield could be considered for the increment of aroma.

## Data Availability Statement

The original contributions presented in the study are included in the article/Supplementary Material, and further inquiries can be directed to the corresponding author.

## Author Contributions

XT initiated and designed the research. HL, MD, LK, LH, YC, and ZW performed the experiments. HL analyzed the data and wrote the manuscript. All authors contributed to the article and approved the submitted version.

## Funding

This study was supported by National Natural Science Foundation of China (31971843), The Technology System of Modern Agricultural Industry in Guangdong (2020KJ105), and Guangzhou Science and Technology Project (202103000075).

## Conflict of Interest

The authors declare that the research was conducted in the absence of any commercial or financial relationships that could be construed as a potential conflict of interest.

## Publisher’s Note

All claims expressed in this article are solely those of the authors and do not necessarily represent those of their affiliated organizations, or those of the publisher, the editors and the reviewers. Any product that may be evaluated in this article, or claim that may be made by its manufacturer, is not guaranteed or endorsed by the publisher.
